# Trans-scaphoid dislocation of the proximal row: a case report

**DOI:** 10.11604/pamj.2013.15.16.2161

**Published:** 2013-05-08

**Authors:** Mohammed Tahir Ansari, Prakash Kotwal, Laxman Rijal

**Affiliations:** 1 All India Institute of medical sciences, New Delhi, India; 2 Department of Orthopaedics, Civil Service Hospital, Minbhawan, Kathmandu, Nepal, India

**Keywords:** Trans-scaphoid dislocation, CT scan, carpectomy, wrist score

## Abstract

This report is of a 32 year old man who presented with complains of pain, swelling and deformity of right wrist of four weeks duration. He gave history of road traffic accident four weeks back leading to injury to right wrist; Preoperative radiographs and C.T. scan images were suggestive of trans-scaphoid dislocation of the proximal row of wrist. A volar and dorsal approach were used to reduce this complex dislocation but was not successful. Wrist arthodesis was performed after doing proximal row carpectomy. One year follow-up of the patient showed fair result with grip strength of 85% to contralateral side and modified Mayo wrist score of 65 at one year.

## Introduction

Dislocation of wrist is severe injury resulting from extreme position of the wrist. Trans-scaphoid perilunate dislocation is the most common dislocation pattern.[1] Isolated dislocation of carpal bone has been described of which lunate dislocation is the most common isolated carpal bone dislocation. Rare patterns involve the dislocation of the scaphoid [2] pisiform [3–6] triquetrum [7] trapezium [8] trapezoid [9] capitate [10] hammate [11] and scapholunate[12] as a unit. Dislocation of the proximal row is not described in English literature. The closest injury pattern described is transverse fractures of the scaphoid, triquetrum, lunate, and pisiform with volar dislocation to the mid-forearm [13].

## Patient and observation

**Figure 1:**
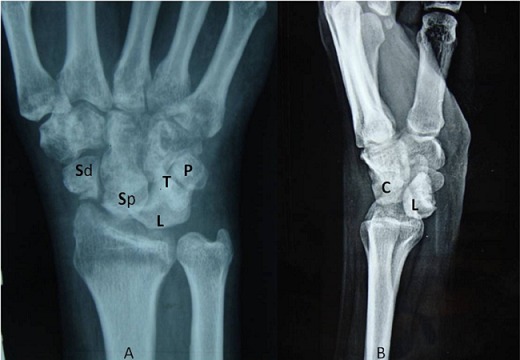
Radiographs showing (a) dislocated proximal pole of scaphoid (Sp), lunate(L), triquetrum(T) and pisiform(P) in PA view, (b) showing volar subluxation of the lunate in lateral view

**Figure 2:**
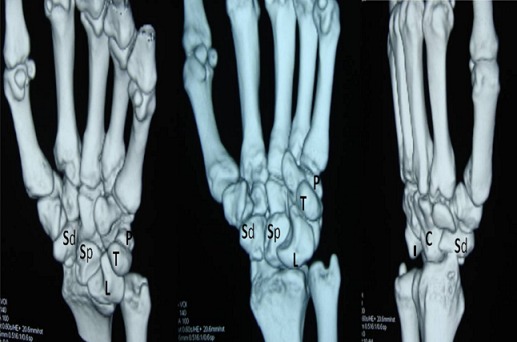
(a),(b),(c): Computed tomography 3D images showing dislocated proximal row scaphoid (Sp), lunate(L), triquetrum(T) and pisiform(P) pushing the capitulam(C)

**Figure 3:**
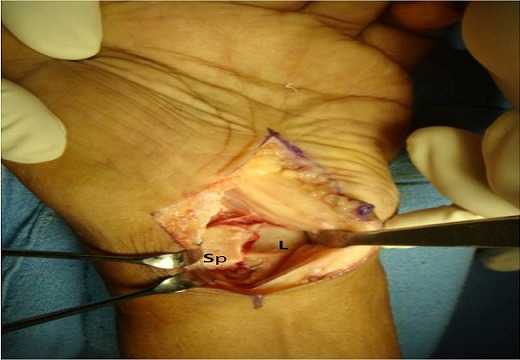
Intraoperative photograph showing dislocated Proximal pole scaphoid (Sp) and lunate(L)

**Figure 4:**
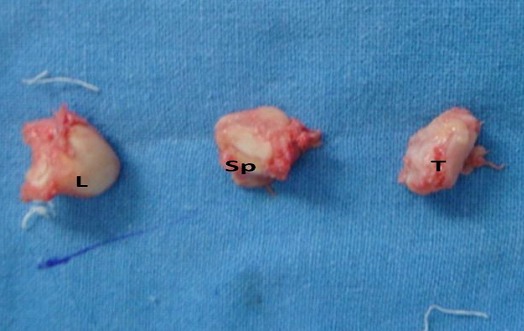
Surgically removed proximal fragment of scaphoid (Sp), lunate (L) and triquetrum (T)

**Figure 5:**
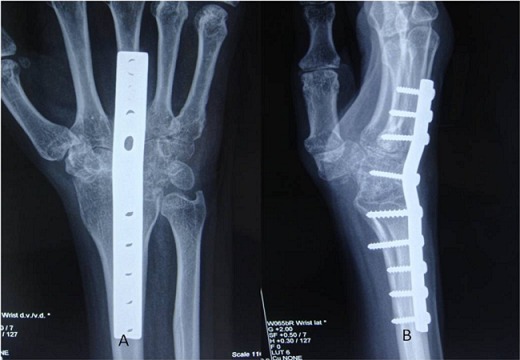
AP (a) and Lateral (b) radiographs showing sound union at one year follow up

A thirty two year old right hand dominated male patient presented with complaints of swelling, pain on movements, limited movements, and loss of grip strength of right wrist of four week‘s duration. The patient had a road traffic accident four weeks back leading to high velocity injury and fall on the outstretched hand with wrist in dorsiflexion and radial deviation. The patient consulted a local practioner who treated him in a plaster of Paris slab for two weeks. The slab was removed after two weeks, the patient noticed a decrease in the swelling but continued to have pain, limited movements and loss of grip strength for which he was referred to our hospital which is the Apex centre of the country. The consulted four weeks after the injury for which x rays (Figure 1) were done. The x-rays, done four weeks after the injury, were suggestive of fracture scaphoid and dislocation of the proximal row of carpals. C.T. scan (Figure 2) was done after reviewing the X-rays which confirmed the diagnosis. The patient was advised proximal row carpectomy or wrist arthodesis if reduction of carpals failed since the injury was old. The patient was taken for surgery under guarded prognosis with consent for wrist arthrodesis if open reduction fails. Volar and dorsal approaches were used to open the wrist joint. The proximal pole of scaphoid, lunate, triquetrum and pisiform were found to be dislocated as a unit (Figure 3). Decompression of the carpal tunnel was done and reduction of the proximal row was attempted but failed. Dislocated proximal row was excised (Figure 4) and wrist arthodesis was performed with the wrist in neutral position. The excised carpal bones were crushed and used as bone graft. The patient was mobilised two weeks after the surgery and was followed for one year. The follow-up x-rays showed sound union (Figure 5). The patient showed fair result with grip strength of 85% to contralateral side and modified Mayo wrist score [14] of 65 at one year.

## Discussion

The wrist is relatively a rare joint to dislocate due to strong volar and dorsal ligaments and the tough attachment of the capsule. Most of the variations of wrist dislocation occur around the lunate as lunate being the most tightly bound carpal to the radius. In 1980, Mayfield staged a series of experiments; the results of these experiments led him to develop a new classification system of perilunate dislocations [15]. The present case will be among the Mayfield stage four of perilunate dislocations in which the lunate subluxates volarly. The major difference between this case and previously reported cases is that the proximal pole of scaphoid, lunate, triquetrum and pisiform dislocated volarly as a unit. The most common pattern among the perilunar dislocations is the stage one or the disruption of scapholunate ligament which was surprisingly intact in this case as the scaphoid fractured at its waist. Despite the early diagnosis and treatment results still remain dependent upon the time of intervention. About 25% of the cases of most common dislocation pattern are missed. The rare patterns of injuries are definitely more prone to be missed due to lack of careful evalution of the roentograms. Any dislocation pattern of carpal bones is an emergency and should be reduced after providing adequate analgesia by haematoma block or general anaesthesia. Delay in treatment affects the outcome of treatment. Our case presented after four weeks of injury so it was expected that the reduction would be difficult, hence he was given an option of wrist arthodesis or proximal row carpectomy. The patient was a manual worker and hence he opted for wrist arthodesis. One year after the surgery he has fared well.

## Conclusion

We conclude that the rare dislocation pattern as this case was, presents as a challenge to orthopaedic surgeon and diagnosis should not be missed, as later presentation leads to difficulty in reduction. Salvage procedures such as wrist arthodesis and proximal row carpectomy are the good alternative procedures with fair results.
